# Current experience with applying the GRADE approach to public health interventions: an empirical study

**DOI:** 10.1186/1471-2458-13-9

**Published:** 2013-01-08

**Authors:** Eva A Rehfuess, Elie A Akl

**Affiliations:** 1 Institute for Medical Informatics, Biometry and Epidemiology, University of Munich, Munich, Germany; 2 Department of Medicine, State University of New York at Buffalo, Buffalo, NY, USA; 3 Department of Clinical Epidemiology and Biostatistics, McMaster University, Hamilton, ON, Canada; 4 Department of Internal Medicine, American University of Beirut, Beirut, Lebanon

## Abstract

**Background:**

The Grading of Recommendations Assessment, Development and Evaluation (GRADE) approach has been adopted by many national and international organisations as a systematic and transparent framework for evidence-based guideline development. With reference to an ongoing debate in the literature and within public health organisations, this study reviews current experience with the GRADE approach in rating the quality of evidence in the field of public health and identifies challenges encountered.

**Methods:**

We conducted semi-structured interviews with individuals/groups that have applied the GRADE approach in the context of systematic reviews or guidelines in the field of public health, as well as with representatives of groups or organisations that actively decided against its use. We initially contacted potential participants by email. Responses were obtained by telephone interview or email, and written interview summaries were validated with participants. We analysed data across individual interviews to distil common themes and challenges.

**Results:**

Based on 25 responses, we undertook 18 interviews and obtained 15 in-depth responses relating to specific systematic reviews or guideline projects; a majority of the latter were contributed by groups within the World Health Organization. All respondents that have used the GRADE approach appreciated the systematic and transparent process of assessing the quality of the evidence. However, respondents reported a range of minor and major challenges relating to complexity of public health interventions, choice of outcomes and outcome measures, ability to discriminate between different types of observational studies, use of non-epidemiological evidence, GRADE terminology and the GRADE and guideline development process. Respondents’ suggestions to make the approach more applicable to public health interventions included revisiting terminology, offering better guidance on how to apply GRADE to complex interventions and making modifications to the current grading scheme.

**Conclusions:**

Our findings suggest that GRADE principles are applicable to public health and well-received but also highlight common challenges. They provide a starting point for exploring options for improvements and, where applicable, testing these across different types of public health interventions. Several public health organisations are currently testing GRADE, and the GRADE Working Group is eager to engage with these groups to find ways to address concerns.

## Background

Public health interventions affect large population groups and can generate significant health benefits at individual and population levels. Even though many public health approaches are preventive in nature, intervening in people’s lives may nevertheless do harm as well as good. In addition, they consume both financial and human resources, and may compromise individual freedom of choice. Public health interventions range from programmatic activities that initiate direct, proximal changes in a specific technology or behaviour to those that bring about more distal changes in multi-sectoral policies with indirect impacts on health [1]. These interventions often combine several approaches that are designed by and delivered through the health sector and/or other sectors [2].

Evaluating public health interventions is far from straightforward and there is much discussion as to how evidence should be gathered, synthesised and used in decision making [3-9]. Developing recommendations or policies in public health relies on complex judgements about a range of factors including magnitude of the health problem, benefits and harms of a given intervention, use of personnel and financial resources, transferability, as well as intervention acceptability and feasibility. Making the decision-making process explicit and transparent is critical, as is a careful examination of the types of evidence underlying specific judgements and, in particular, the quality of evidence in support of likely benefits and harms.

Public health organisations in different countries have developed distinct approaches to convey the quality of the evidence [10].These include the *Guide to Community Preventive Services* (Community Guide) issued by the United States Community Preventive Services Task Force [11,12], Public Health Guidance developed by the National Institute for Health and Clinical Excellence (NICE) [13] and the Netherland’s Organisation for Public Health’s recognition system for health promotion interventions [14,15]. While, to our knowledge, these have not been formally compared, the use of many different schemes in parallel may lead to a divergent rating of the quality of evidence and conflicting recommendations. This may hinder the goal of helping guideline developers and policy-makers make well-informed decisions in a transparent way, both nationally and internationally [16].

The Grading of Recommendations Assessment, Development and Evaluation (GRADE) Working Group, a network of primarily clinical guideline developers and systematic reviewers, has attempted to meet this challenge by developing and testing a rigorous, systematic and transparent framework for evidence-based guideline development [17-19]. In a first step, the quality of the evidence (defined as the extent of our confidence that the estimate of effect is correct and/or that this estimate is adequate to support a particular recommendation) is classified in one of four categories: high, moderate, low or very low quality [20,21]. Randomised controlled trials begin as high quality, while observational study designs (including non-randomised or quasi-randomised intervention studies as well as cohort studies, case control studies and other correlational study designs) begin as low quality. The quality of the evidence can subsequently be rated down based on five criteria (i.e. risk of bias, inconsistency, indirectness, imprecision, publication bias) or rated up based on three criteria (i.e. strong association, dose-response gradient, plausible confounding). In a second step, the strength of a recommendation (defined as the extent to which we can be confident that desirable effects of an intervention outweigh undesirable effects) is graded as either strong or weak (conditional or discretionary). This judgement of the strength of a recommendation is based on magnitude of desirable and undesirable consequences, quality of evidence, values and preferences and resource use.

More than 65 national and international organisations have adopted the GRADE approach (see http://www.gradeworkinggroup.org/society/). While the GRADE Working Group promotes the use of the framework across clinical and non-clinical health evidence, there has been much debate in the literature [22-28] and within public health organisations (e.g. European Centre for Disease Prevention and Control, Swedish National Institute of Public Health, Canadian Public Health Agency, World Health Organization) as to whether this scheme is well-suited to public health interventions.

As a contribution to this debate, the objectives of this study were to review current use of and experience with the GRADE approach in rating the quality of evidence in the field of public health, and to identify challenges encountered.

## Methods

We conducted semi-structured interviews with two groups: individuals that have applied the GRADE approach in the context of systematic reviews or guidelines in the field of public health and selected individuals representing public health organisations that actively decided against the use of the GRADE approach. Both groups, given their familiarity with the GRADE approach, are in principle in a position to report specific challenges encountered, with semi-structured interviews providing room for open, in-depth feedback. In addition, we attempted to document whether organisations known to develop evidence-based public health guidance have adopted the GRADE approach or not, although this was not undertaken in a systematic way.

Public health interventions were classified *a priori* as (i) health policy, (ii) health system (iii) behavioural, (iv) nutrition, (v) environmental, (vi) vaccination and (vii) screening interventions. *Post hoc* we also added the category clinical interventions, as several answers provided by respondents related to treatment guidelines.

We primarily identified potential participants through the recommendations of members of the GRADE Working Group and the Guidelines Review Committee at the World Health Organization. We also approached the Cochrane Collaboration’s Public Health Group, the Cochrane Collaboration’s Effective Practice and Organisation of Care Group, the Campbell Collaboration, the SUPPORT collaboration through the Norwegian Knowledge Centre for the Health Services, the Public Health Agency of Canada, the Swedish National Institute of Public Health, the United States Community Preventive Services Task Force, the European Centre for Disease Prevention and Control and the NICE Centre for Public Health Excellence in the United Kingdom.

We initially contacted potential participants by email with up to two email reminders as needed, inviting them to share their experience with applying the GRADE approach. We asked respondents the following three open-ended questions, and encouraged them to provide specific examples to illustrate their responses:

• What are the challenges encountered in applying the GRADE approach to rating evidence for public health interventions?

• Does the evidence grade obtained through GRADE adequately reflect the confidence in the evidence?

• Could fine-tuning of the existing rating of evidence make the GRADE approach more applicable to public health interventions?

In most cases, answers were obtained by telephone interview; in the remaining cases, answers were summarised based on email correspondence. We established experience with the use of GRADE for each interview as (i) applied without significant challenges, (ii) applied with minor challenges, (iii) applied with major challenges, (iv) currently being considered/tested, and (v) not currently applied. Specific challenges encountered and suggestions to change the existing rating of the evidence were initially identified by ER from written, validated summaries of individual interviews. These were then analysed across individual interviews by ER and EA to distil broader common themes.

In order to validate our findings, we asked each participant to review and correct written summaries of the interviews and our rating of her/his experience with the application of GRADE. As necessary, we approached participants up to three times for validation. At a later stage, we also shared the draft paper with all participants and a wide range of interested parties to obtain feedback on our interpretation of findings. The study was acknowledged by the ethics committee at the University of Munich; ethics approval was not required.

## Results

Of 29 individuals/groups in 12 organisations that were approached, 4 did not respond. Table 1 lists the organisation, the group within the organisation, the rating of their use of GRADE or lack of GRADE endorsement, and the number of in-depth responses. 15 interviews were undertaken with individuals/groups that had applied GRADE in one or more systematic review or guideline projects and provided in-depth responses on their experiences (Additional file 1). An additional 3 interviews were conducted with individuals/groups that were familiar with GRADE but actively decided against using the approach. The remaining 7 responses were recorded but did not lead to in-depth interviews (Table 1).

**Table 1:** Overview of organisations approached and responses obtained

**Organisation**	**Group**	**# responses**	**GRADE application to public health interventions**	**# specific responses contributing to sample**
**Cochrane Collaboration**	Effective Practice and Organisation of Care Group	1	GRADE applied without significant challenges	0
	Public Health Group	1	GRADE endorsed and currently being tested	1
**Campbell Collaboration**	Methods Group	1	GRADE not currently applied	0
**World Health Organization**	HIV/AIDS	1	GRADE applied without significant challenges	1
	Tuberculosis	1	GRADE applied with minor challenges	1
	Nutrition for Health and Development	1	GRADE applied without significant challenges	1
	Maternal, Newborn, Child and Adolescent Health	2	GRADE applied with minor/major challenges	2
	Immunisation, Vaccines and Biologicals	1	GRADE applied with minor challenges	1
	Mental Health	1	GRADE applied with major challenges	1
	Public Health and Environment	2	GRADE applied with major challenges	1
	Health Systems and Services	1	GRADE applied with minor challenges	1
	Reproductive Health and Research	1	GRADE applied with minor/major challenges	1
**Public Health Agency of Canada**	Centre for Chronic Disease Prevention and Control	1	GRADE currently being tested	0
**Canadian Guidelines for Immigrant Health**	-	1	GRADE applied with minor challenges	1
**Canadian Task Force on Preventive Health Care**	-	1	GRADE applied with major challenges	1
**Swedish National Institute of Public Health**	Department of Child and Elderly Health	1	GRADE endorsed and currently being tested	0
**United States Community Preventive Services Task Force**	Centers for Disease Control and Prevention, Community Guide Branch	1	GRADE not currently applied	0
**European Centre for Disease Prevention and Control**	Office of the Chief Scientist	1	GRADE not currently applied	0
**National Institute for Health and Clinical Excellence, United Kingdom**	Centre for Public Health Excellence	1	GRADE not currently applied	0
**Norwegian Knowledge Centre for the Health Services**	SUPPORT network	3	GRADE applied with minor challenges	2
**Boston University**	Centre for Psychiatric Rehabilitation	1	GRADE not currently applied	0
**Total**	**-**	**25**	**-**	**15**

Minor challenges: no major concerns, challenges in relation to exact interpretation of GRADE criteria and challenge with respect to GRADE application.

Major challenges: major concerns, introduction of new GRADE criteria, use of observational and non-epidemiological evidence.

### What are the challenges encountered in applying the GRADE approach to rating evidence for public health interventions?

Detailed responses to this question among the individuals/groups who had applied GRADE in one or more systematic review or guideline projects (Additional file 1) can be summarised as GRADE is applied without significant challenges (2 responses), GRADE is applied with minor or major challenges (12 responses) and GRADE is currently being tested (1 response) (Table 1). As is illustrated in Table 2, GRADE has been applied across a range of public health interventions.

**Table 2:** Overview of specific topics covered, classified by intervention type

**Type of intervention**	**Topics covered**
**Health policy interventions**	SUPPORT summary: Do pharmaceutical reference and index pricing policies have effects on drug use, health care utilisation, health outcomes and costs?
**Health systems interventions**	WHO guideline on prevention and treatment of HIV and other sexually transmitted infections among men who have sex with men and transgender people
	WHO guideline on retention of rural health workers
	Various WHO guidelines on community-based newborn care
	SUPPORT summary: Do conditional cash transfers improve the uptake of health interventions in low and middle-income countries?
	SUPPORT summary: Does pay-for-performance improve the quality of health care?
	SUPPORT summary: Does expanding the role of outpatient pharmacists improve healthcare delivery and patient outcomes?
	SUPPORT summary: Does prompting physicians improve performance in preventive care?
**Nutrition interventions**	Guideline: Neonatal vitamin A supplementation
	Guideline: Vitamin A supplementation for infants 1–5 months of age
	Guideline: Vitamin A supplementation for infants and children 6–59 months of age
	Guideline: Vitamin A supplementation in pregnant women
	Guideline: Vitamin A supplementation in postpartum women
	Guideline: Vitamin A supplementation during pregnancy for reducing the risk of mother-to-child transmission of HIV
	Guideline: Intermittent iron supplementation in preschool and school-age children
	Guideline: Intermittent iron and folic acid supplementation in menstruating women
	Guideline: Daily iron and folic acid supplementation in pregnant women (in press)
	Guideline: Intermittent iron and folic acid supplementation in non-anaemic pregnant women (in press)
	Guideline: Use of multiple micronutrient powders for home fortification of foods consumed by infants and children 6–23 months of age
	Guideline: Use of multiple micronutrient powders for home fortification of foods consumed by pregnant women
	WHO recommendations for prevention and treatment of pre-eclampsia
	WHO guidelines on HIV and infant feeding
**Behavioural interventions**	Canadian physical activity and sedentary guidelines for the early years
**Environmental interventions**	WHO indoor air quality guidelines: household fuel combustion (ongoing)
	Cochrane systematic review on housing improvements for health and associated socioeconomic outcomes (ongoing)
**Vaccination**	Cholera vaccines: WHO position paper
	Hepatitis B vaccines: WHO position paper
	Human papillomavirus vaccines: WHO position paper
	Measles vaccines: WHO position paper
	Meningococcal vaccines: WHO position paper
	Pertussis vaccines: WHO position paper
	Pneumococcal vaccines: WHO position paper
	Polio vaccines and polio immunization in the
	pre-eradication era: WHO position paper
	Rabies vaccines: WHO position paper
	Rubella vaccines: WHO position paper
	Vaccines against tick-borne encephalitis: WHO position paper
**Screening interventions**	Guidelines on cervical cancer screening
	Guidelines on breast cancer screening
	Guidelines on latent TB screening among immigrants and refugees
	Guidelines for hepatitis B screening among immigrants and refugees
**Clinical interventions**	WHO guidelines for the programmatic management of drug-resistant tuberculosis
	WHO guidelines for mental health, neurological and substance use disorders within WHO Mental Health Gap Action Programme
	WHO guidelines on prevention and control of cervical cancer (ongoing)
	WHO guidelines on the management of children with severe malnutrition

Two individuals/groups reported no significant challenges in applying GRADE. They emphasised that it offers an explicit, transparent, rigorous and flexible process that forces systematic reviewers and guideline developers to consider limitations in the evidence base. It brings objectivity to the guideline development process from formulating the question to appraising evidence to deriving a recommendation.

Twelve individuals/groups reported challenges in applying GRADE. More than half of them described these challenges as minor (i.e. challenges in relation to exact interpretation of GRADE criteria, challenges with respect to GRADE application), the other half suggested that these were major (i.e. introduction of new GRADE criteria, use of observational and non-epidemiological evidence). Even among the groups reporting challenges general feedback about the GRADE approach was positive. Users emphasised that GRADE principles apply to public health interventions, appreciated the systematic and transparent process of assessing the quality of evidence and stated that the GRADE approach has improved the quality of guideline development.

Specific challenges reported can be grouped as follows:

• *Complexity of public health interventions:* Public health interventions tend to be complex with several components. In principle, studies assessing these interventions may look at one, all or different combinations of these components although, in practice, most evaluations focus on the impact of the whole package. Guideline developers and systematic reviewers developing a PICO (i.e. population, intervention, comparison, outcome) question for a specific intervention need to either consider the intervention as a whole or focus on a presumed active component. Either decision presents challenges regarding which studies to include or exclude, how to interpret heterogeneity, and how to make a careful judgement about the degree of indirectness.

• *Choice of outcomes and outcome measures:* Most respondents emphasised that the choice of outcomes and outcome measures tends to be complicated in systematic reviews of public health interventions due to (i) multiple outcomes for most interventions, (ii) outcomes at individual and group levels, (iii) reliance on short-term surrogate rather than morbidity and mortality outcomes that are significantly delayed into the future, (iv) inconsistencies associated with the use of competing measures or assessment scales and (v) the need to group heterogeneous measures under “umbrella outcomes” to make the review policy-relevant. The choice of outcome and outcome measures has implications for indirectness of evidence and needs to be decided on and judged carefully as part of the GRADE process.

• *Ability to discriminate between different types of observational studies:* Many groups reported difficulty using GRADE to rate public health evidence derived from observational studies. With the quality of evidence for all types of non-randomised studies starting as low, the GRADE approach is perceived as lacking the ability to distinguish between those public health interventions that are reasonably well-supported by evidence (e.g. by interrupted time series studies) and those that are less supported by evidence (e.g. by cohort studies). This may lead to misinterpretations of the evidence when communicating the message to policy-makers, and may even discourage the conduct of “best possible” studies. The GRADE approach should encourage people to develop and make the best use of high-quality evidence and not lead to an unrealistic restriction of the evidence, even if unintended.

• *Use of non-epidemiological evidence:* Several groups also noted that the GRADE approach does not allow users to integrate non-epidemiological evidence, such as laboratory, mechanistic or animal studies, the principles of other disciplines (e.g. physiology, engineering, toxicology, chemistry, physics) and evidence on implementation and context. All of these can be critical when assessing the effectiveness of a public health intervention (e.g. as individuals are exposed to air pollution indoors as well as outdoors, a whole-village implementation approach for improved cookstoves will show much greater reductions in household air pollution and respiratory health outcomes than a single-household implementation approach), as well as when making a recommendation concerning implementation. As one respondent put it, ”relying solely on epidemiological evidence to assess intervention effectiveness is imprudent, as there are many factors influencing the results beyond whether or not the principle of a technology works.”

• *GRADE terminology:* Several groups stated that some of the GRADE terminology and definitions were not appropriate for public health interventions (e.g. definition of quality of evidence, use of the terms patients and clinicians). They also expressed concern about possible misinterpretations by policy-makers of “low quality evidence” and/or “weak recommendations”, with such wording potentially being used to justify inaction. Likewise, GRADE uses the term “observational study” to refer to all studies that are not randomised. In the public health world, the major distinction tends to be between observational studies where the assignment of individuals/clusters to an exposed/intervention group *vs.* unexposed/control group is outside of the control of the investigator (e.g. case–control, cohort, cross-sectional, patient series, time series) and those where the assignment of individuals/clusters to an intervention *vs.* control group is undertaken by the investigator in a randomised or non-randomised fashion (e.g. individually randomised, cluster-randomised trials, quasi-/pseudo-randomised trials, controlled before-and-after studies). Possibly as a result of this confusion in terminology some guideline projects have quasi-experimental designs begin as high followed by downgrading rather than having these begin as low followed by upgrading, as recommended by the GRADE approach.

• *GRADE and guideline development process:* Several respondents mentioned that GRADE is often applied to systematic reviews that do not consider all relevant data, in part because including non-randomised study designs makes the systematic review process methodologically challenging and much more time-consuming. Where the results of a systematic review are very heterogeneous and effect estimates cannot be pooled, applying GRADE criteria (e.g. inconsistency, imprecision, magnitude of effect, publication bias) to narrative estimates of impact becomes challenging. Some respondents also noted that the GRADE approach was not sufficiently objective and reproducible. Many respondents suggested that the GRADE process for going from evidence to recommendations was both difficult and *ad hoc* in terms of taking into consideration values and preferences, cost effectiveness and feasibility issues (e.g. characteristics of delivery agents or systems, availability of implementation studies). Finally, there were concerns about the major financial and human resources required.

The United States’ Community Guide and the Public Health Guidance offered by the United Kingdom’s NICE Centre for Public Health Excellence are often referred to as models for evidence-based public health decision-making. Both organisations explicitly decided against using the GRADE approach:

"· We perceive challenges in applying GRADE to the types of evidence we commonly use in the development of recommendations – these often include observational studies for questions of effectiveness and other types of studies for answering a range of other questions, such as qualitative research about the views of service users. We also use observational studies to help identify potential barriers to implementation. Further, as well as considering the quality, strength and applicability of the evidence, our committees take into account cost effectiveness, ethics, equity and other issues in developing recommendations. We think our current system gives us the flexibility we need, given the breadth of public health topics, the types of questions and the range of outcomes considered.” (Simon Ellis, NICE Centre for Public Health Excellence, United Kingdom)"

"· At the Community Guide we do not use GRADE because we do not believe it works well when applied to population-level interventions. Specifically, it does not adequately differentiate between quasi-experimental designs with differing degrees of protection against threats to internal validity, and hence undervalues the evidence from the strongest of these designs. It is particularly problematic when there are many studies for which the most plausible threats to validity are expected to be randomly distributed, rather than systematically biasing the results in one direction.“ (Randy Elder, Community Guide Branch, United States Centers for Disease Control and Prevention)"

Similarly, having explored its use, the Center for Psychiatric Rehabilitation at Boston University actively rejected the GRADE approach:

"· Interventions in the field of psychiatric disability and mental illness are characterised by complexity, as they critically rely on interactions between two or more human beings, as well as social groups. In view of small sample sizes available, the typical course of exacerbations and remissions for many mental illnesses, and issues associated with role performance and societal integration outcomes, RCTs are very difficult to conduct. Therefore, the majority of studies in our field, with some notable exceptions, have been correlational, observational or quasi-experimental to this point. Given that GRADE, from the outset, determines observational studies to be of low quality, we felt that we had to begin from the question – what standards would properly assess such studies and their findings in terms of scientific rigor and meaning, rather than assessing them against a standard that they would rarely be able to meet. As a result, we developed our own grading systems for such studies.” (Marianne Farkas, Center for Psychiatric Rehabilitation, Boston University)"

### Does the evidence grade obtained through GRADE adequately reflect the confidence in the evidence?

Most respondents felt that the evidence grade obtained by applying GRADE to their systematic review or guideline project was appropriate. They stated that the application of the GRADE criteria had opened their eyes to important limitations in the underlying evidence base. At the same time, they expressed the following concerns:

• *Meaning of confidence in the evidence:* GRADE defines quality of evidence as confidence in the pooled effect estimate. As the effectiveness of public health interventions is critically influenced by modes of delivery and contextual issues, a more relevant interpretation would be “confidence that the effect is meaningful across a range of plausible implementation contexts” (Randy Elder, Community Guide Branch, United States Centers for Disease Control and Prevention)

• *Selection of the appropriate body of evidence for rating can be difficult:* Several groups stated that it is challenging to grade the quality of evidence, in the presence of both a single or few randomised studies (rated as high quality, not downgraded for inconsistency or reporting bias) and a number of non-randomised studies (rated as low quality, potentially downgraded for inconsistency and reporting bias). In the extreme case, can one well-conducted and large RCT conducted in one setting and rated as high-quality be sufficient to decide on the effectiveness of a complex intervention that critically relies on context?

•*All observational study designs begin as low-quality evidence:* A number of participants stated that the current approach does not appropriately discriminate between stronger and weaker observational study designs. They suggested that quasi-experimental designs, interrupted time series studies and self-controlled case series should begin as moderate quality. In fact, some respondents felt that applying the GRADE criteria resulted in multiple discriminations against observational studies, where these studies started off low and were at risk of being penalised once more for threats to validity inherent to their design.

•*There are insufficient possibilities for upgrading observational studies:* Different groups suggested a broader interpretation of existing criteria, such as extending the concept of dose–response to the population level, and the addition of new criteria for upgrading, in particular consistency and analogy. While GRADE allows for the downgrading of a body of evidence for inconsistency, consistency in findings across different settings, study designs and research groups actually increases our confidence in the evidence, as already stated by Sir Austin Bradford Hill. Analogy extends the concept of consistency to parallel evidence from related risk factors, interventions or population groups.

•*Non-epidemiological evidence can only be rated as very low-quality:* Judging the effectiveness of a public health intervention sometimes relies on sources of evidence outside of epidemiology. Physiological, physical or engineering principles and the insights gained through laboratory or animal studies can only be brought into the rating exercise as a separate very low-quality piece of evidence rather than lending additional credibility to epidemiological evidence. GRADE also lacks a structured approach for integrating evidence on implementation and context, issues that often directly impact the effectiveness of complex interventions.

### Could fine-tuning of the existing rating of evidence make the GRADE approach more applicable to public health interventions?

As has been apparent in previous sections, respondents did not limit their comments to rating the quality of evidence but provided feedback on the GRADE approach overall. Here, we focus on suggestions for fine-tuning the rating step; more general feedback has already been reported above. Suggestions by respondents largely fall into two categories and reflect comments discussed above: pragmatic guidance on the application of the GRADE approach for complex interventions and modifications to the existing GRADE criteria.

Respondents expressed uncertainty about and requested pragmatic guidance on:

• How to reflect intervention complexity in rating quality of evidence, in view of much of the available primary studies and systematic reviews simplifying intervention complexity;

• How to prioritise individual *vs.* group level outcomes, short term *vs.* longer term outcomes and surrogate *vs.* morbidity and mortality outcomes, as differences in prioritisation are likely to impact the evidence rating;

• How to decide whether very limited randomised evidence is sufficient or not, as including non-randomised evidence will have an impact on how the quality of evidence is rated;

• How to ensure reproducibility in rating the quality of evidence, both in relation to individual GRADE criteria and their integration; and

• How to apply the GRADE criteria to narrative summaries.

Respondents also proposed specific modifications to rating the quality of evidence:

• That selected observational study designs start off as moderate in order to discriminate more accurately between different levels of internal validity;

• That additional criteria for upgrading be developed and operationalised, as these would allow confidence in a body of observational evidence to be reflected more accurately;

• That a systematic approach for incorporating non-epidemiological evidence that directly relates to intervention effectiveness be developed; and

• That a systematic approach for assessing the quality of modelling studies be considered.

## Discussion

### Key findings

The objective of this study was to review the current use of and accumulating experience with the GRADE approach in rating the quality of evidence in the field of public health, and to identify challenges encountered to date. Despite the challenges reported, it is important to note that all respondents that have employed GRADE in a systematic review or guideline project appreciated the systematic and transparent process of assessing the quality of evidence. This highlights that the general principles of the approach are both suitable to public health and well-received.

Common themes emerged with respect to aspects of the GRADE approach in its current form lacking applicability, reproducibility and clarity in the field of public health. Specific challenges related to (i) complexity of public health interventions, (ii) choice of outcomes and outcome measures, (iii) ability to discriminate between different types of observational studies, (iv) use of non-epidemiological evidence, (v) GRADE terminology and (vi) the GRADE and guideline development process. Respondents also made suggestions for additional pragmatic guidance and specific modifications to rating the quality of evidence; these are integrated in the below proposal towards making the GRADE approach more applicable to public health interventions.

Several of the challenges reported are not specific to GRADE *per se* but relate more to the process of systematically reviewing and synthesising complex public health evidence; by requiring a transparent and structured approach for rating the quality of evidence and grading the strength of a recommendation, GRADE makes these challenges salient. In particular, the guideline development process often relies on systematic reviews that do not consider all relevant data, that are based on very heterogeneous data and that conclude with narrative syntheses rather than meta-analyses. Some of these issues are revisited in more detail below. Also, while it is argued that use of the GRADE approach substantially increases financial and human resource costs, a majority of these additional resources is likely to be required for conducting systematic reviews rather than for the application of GRADE.

It is noteworthy that the majority of specific GRADE applications in the field of public health to date appear to have taken place at the World Health Organization where the use of GRADE has been mandatory since 1 January 2009. GRADE is also used as part of the SUPPORT Collaboration and at the Norwegian Knowledge Centre for the Health Services, which acts as one of the satellites for the Cochrane Collaboration’s Effective Practice and Organisation of Care Group. This rather limited experience with GRADE in the public health field begs the question whether this research was conducted ahead of its time, or whether the added value of the GRADE approach to public health beyond current practices is not sufficiently apparent. The fact that several groups are currently testing GRADE (Table 1) and that some of those groups who explicitly decided against use of GRADE expressed an interest in the results of this study and are, in principle, willing to revisit their decision suggests that this study is timely.

### Complexity of public health interventions revisited

Several respondents reported difficulties in applying GRADE that relate to complexity of public health interventions, e.g. their proactive, multi-component nature, a focus on the population level, a multiplicity of relevant outcomes and influence of implementation mode and context on effectiveness. Table 3 suggests an extended PICO approach that systematically notes key differences between “simple” and “complex” interventions. Please note that we avoid a distinction between clinical and public health interventions, as both types of interventions tend to be located along a spectrum of complexity, i.e. many of the concerns are not unique to public health interventions but equally apply to complex clinical interventions.

**Table 3:** Comparison of “simple” and “complex” interventions

	**“Simple” interventions**	**“Complex” interventions**
**Population**	Sick population seeking care	Healthy general or at-risk population
**Intervention**	Individual-level intervention	Population- and/or individual-level intervention
	Single component	Multiple interacting components
	“Reactive” treatment through medication or surgery or clinical prevention	“Proactive” prevention through behaviour change and/or technical intervention and/or policy
	Implementation in healthcare setting	Implementation in household, community or policy setting
**Comparison**	No intervention or alternative intervention through treatment/surgery	“Business as usual” in several sectors
**Outcome**	Shorter causal pathway	Longer causal pathway
	One or a small number of health outcomes	Multiple health outcomes and broader societal consequences
	Usually impact after short lag period	Usually impact after long lag period
**Delivery of intervention**	Delivery through health sector	Delivery through multiple sectors
**Contextual effects**	Variation between healthcare providers (individuals, institutions)	Variation between providers of different intervention components in multiple sectors
	Patient preference and compliance	Large cultural and behavioural variation

“Simple interventions” tend to show more of the characteristics in the left column while “complex” interventions tend to show more of those in the right column.

There is increasing recognition that complex preventive or curative interventions should be developed and tested using a theory-driven, phased approach [3,29-31], which somehow mimics the sequential phases of drug development. To date, however, public health interventions that follow this approach are rare, which means that evidence-based decision-making continues to rely on systematic reviews that evaluate the effectiveness of an amalgam of interventions combining components that vary in number, quality and intensity. Unfortunately, small sample sizes in some categories did not allow us to explore more formally how the GRADE experience varies in relation to intervention complexity, where on average health policy, health systems or environmental health interventions tend to be more complex than clinical, screening or vaccination interventions (Table 2). Yet, it is apparent from Table 3 that a more or less complex interpretation of these interventions will have implications for how systematic reviews are scoped and conducted, and for how their findings are rated; while a simple interpretation of many clinical interventions is commonly performed and often provides adequate insight, a simple interpretation of many public health interventions can be misleading (Rehfuess and Bartram, work in progress).

Several respondents suggested that the GRADE approach does not do justice to the insights to be gained from well-conducted non-randomised studies. These are part and parcel of public health evidence, as sometimes RCTs are either not feasible (e.g. pricing of tobacco products, advertising bans for alcohol) or not appropriate where an intervention has multiple or indirect health impacts, where impacts materialise in a gradual or delayed fashion, and where an intervention is so complex that RCT results will be unacceptably artificial [5,32]. Indeed, natural experiments are increasingly recommended as a suitable way of understanding the health impacts of large-scale population health interventions [4]. One respondent went as far as describing a “three-level discrimination against observational study designs” throughout the systematic review and guideline development process: First, those interventions that are difficult to study through RCTs, as described by the “inverse evidence law” (i.e. there tends to be greater quantity and better-quality evidence for simple interventions directed at individuals compared to complex interventions directed at populations [33,34]), are less likely to be the subject of a primary evaluation or a systematic review. Secondly, when systematic reviews are conducted, they often search the literature only for randomised designs, dismissing as noise much of what others would consider to be the signal. Thirdly, when evidence is graded, all observational studies start off as low-quality independent of the greater or lesser internal validity of the specific study design.

Evidence-based public health guidance also needs to draw on sources of evidence beyond the traditional hierarchy of epidemiological study designs. First, public health evidence is never context-free [9], and the importance of capturing information on intervention delivery, economics, equity and the overall socio-economic environment in systematic reviews has been previously emphasised [35]. Secondly, public health is multidisciplinary and multi-sectoral by definition, and thus relies on broader sources of evidence, including “parallel evidence” [36] from related risk factors, interventions or population groups, which may strengthen overall confidence in the quality of the evidence. As of yet, the GRADE approach does not offer a framework for systematically appraising and integrating contextual evidence and evidence generated by disciplines other than epidemiology. A causal-chain approach, where evidence assessments take place across sequential links, where some links are amenable to GRADE in its current form whereas others are not, might offer a starting point (Bruce et al, work in progress).

### Strengths and limitations of study

This study is not representative of all GRADE applications in the field of public health to date. While we approached a large number of organisations and individuals, we may have overlooked others, and therefore potentially missed relevant insights. Although we obtained responses from all organisations approached, some groups or individuals within those organisations did not respond to our three emails to establish initial contact.

Moreover, the characteristics of those interviewed vary greatly, for example in relation to level of epidemiological training, depth and breadth of the GRADE experience (i.e. number of specific GRADE applications and across different GRADE versions) and the level of support received from the GRADE Working Group. As a result, the classification of challenges as minor or major may not be directly comparable. On the other hand, our sample encompasses a range of organisations, health issues (e.g. communicable diseases, non-communicable diseases, health systems) and types of public health interventions, offering the full breadth of experiences to date. Therefore, we believe that this study offers important insights into issues that several organisations and groups are currently struggling with.

While interview summaries and their qualitative interpretation are inherently subjective we had summaries validated and provided all participants with an opportunity to review the draft manuscript. Also, the fact that our empirical approach and the group of experts convened by the European Centre for Disease Prevention and Control [26] identified rather similar challenges is encouraging:

• The GRADE approach is limited to assessing the level of evidence for the efficacy/effectiveness of an intervention, while prioritisation of topics, selection of experts and dealing with potential conflicts of interest should also be handled in a systematic, explicit and transparent manner.

• The GRADE approach employs nomenclature to describe the confidence in the level of evidence that may sound pejorative, such as “low quality”; more neutral terminology would be more appropriate.

• The GRADE approach appears to have too few categories to capture different levels of evidence represented by study designs beyond randomised controlled trials.

• The GRADE approach is very limited in its applicability to other types of evidence, such as microbiological investigations, health economic models, mathematical models of the spread of infectious diseases and incidence/prevalence studies.

• The GRADE approach includes too few criteria for upgrading the quality of observational studies.

• The GRADE approach is limited in its applicability to issues beyond the assessment of efficacy/effectiveness (and possibly safety) of interventions, such as risk assessment, disease causation and spread of infectious diseases.

• The GRADE approach is time-consuming and may thus not be suitable for a rapid grading of evidence under time pressure.

• The GRADE approach does not appear to perform well in situations of scarce evidence that often characterise the field of public health.

• The GRADE approach does not appropriately address going from evidence to recommendations, which should also include considerations of context, law, ethics, economic and political considerations.

### A proposal towards improving GRADE’s applicability to public health interventions

Some groups have shown that finding solutions to many of these challenges within the current version of GRADE is possible [37], others are already implementing some of the proposed changes [22-24]. If GRADE is to remain a standardised approach, it is critical that common issues raised across groups are addressed centrally. Suggestions by respondents largely fall into the categories (i) conceptual and terminology issues, (ii) pragmatic guidance on application of the GRADE approach and (iii) potential modifications to the existing scheme for rating the quality of evidence.

#### Conceptual and terminology issues

We propose that the GRADE Working Group refine and revise, where appropriate, current terminology and definitions to make these more applicable to public health interventions. In particular, this relates to the interpretation of confidence in the evidence, the use of terms such as patients and clinicians *vs.* beneficiaries and practitioners/policy-makers, the definition and classification of study designs, and the terminology used to communicate quality of evidence and strength of recommendation.

Efforts underway to address some of the issues raised include, for example, public health-compatible GRADE guidance on the meaning of outcomes that are critical *vs.* important *vs.* of limited importance, and alternative ways of communicating quality of evidence using symbols such as ⊕⊕⊕Ο [38] or focusing on the likelihood of further evidence to change conclusions [23,24]. The GRADE Working Group has been flexible in the use of terminology to express the quality of evidence (e.g. confidence in effect estimates) and strength of recommendation (e.g. “conditional recommendation” instead of “weak recommendation”).

#### Pragmatic guidance on application of the GRADE approach

We propose that the GRADE Working Group develop pragmatic guidance on how to apply the GRADE approach to complex interventions, illustrated with public health examples. In relation to rating the quality of evidence, guidance should address how to ensure reproducibility and internal consistency, when to consider non-randomised evidence in addition to randomised evidence, and how to apply GRADE criteria to narrative summaries. In relation to going from evidence to recommendations, guidance should include public health-specific considerations that influence the strength of a recommendation.

Current efforts include the DECIDE project, which aims to improve the dissemination of evidence-based recommendations by building on the work of the GRADE Working Group [39]. For example, the DECIDE project has expanded the GRADE framework for moving from evidence to recommendations to include factors such as the prevalence of the problem, incremental cost relative to benefit, equity, feasibility and acceptability. There are also efforts by different groups to make the assessment of value judgements, resource use and feasibility more explicit and transparent [22]. The SUPPORT collaboration offers excellent guidance and worked examples of how GRADE can be applied to narrative summaries [40]. With respect to reproducibility, the GRADE Working Group acknowledges that many steps in the process of rating the quality of evidence are subjective and highlights the importance of transparency in reporting these judgements. It is also currently undertaking studies to assess and improve reliability of the methodology.

#### Potential modifications to the existing scheme for rating quality of evidence

We propose that the GRADE Working Group review whether selected observational study designs should start off as moderate and develop and test additional criteria for upgrading, provided these can be made meaningful and applicable across public health and clinical medicine. The Working Group should also explore how other relevant sources of evidence that do not follow the traditional hierarchy of study designs (e.g. evidence generated by other disciplines, modelling studies) can be integrated with GRADE at the level of rating quality of evidence and/or at the level of grading a recommendation. GRADE should provide more explicit guidance about the use of different types of evidence.

During regular meetings of the GRADE Working Group, specific examples of problems encountered can be raised and discussed, facilitating the continuous evolvement of the GRADE approach. Such a discussion led, for example, to agreement that the dose–response relationship criterion can also be applied at the population level [23,24]. Systematic reviews registered with the Cochrane Collaboration’s Public Health Group and guideline projects at the World Health Organization provide ample opportunity for testing a revised grading scheme.

## Conclusions

This empirical study reviewed current experience with the GRADE approach in the field of public health. It suggests that GRADE principles are applicable to public health, and that the systematic, transparent and rigorous nature of the process is well-received by those groups who have applied it. It also identified several common challenges, particularly in relation to evidence grading, which was the focus of this study. Contributions from respondents suggest that a combination of revisiting concepts and terminology, providing better guidance on how to apply GRADE to complex interventions and making some modifications to the existing scheme for rating quality of evidence could improve the applicability of the GRADE approach to public health interventions. Our proposal is intended as the starting point for a research and development agenda to explore options for improvements and, where applicable, to test these in systematic reviews and guideline projects across different types of public health interventions. Independent of whether the specific challenges identified here are perceived or real, they merit attention, if the GRADE approach is to be more widely adopted in the public health world.

## Competing interests

Both authors are members of the GRADE Working Group.

## Authors’ contributions

ER conceived the study, approached study participants, conducted interviews and assumed primary responsibility for the analysis of findings. EA contributed to the design of the study and the analysis of findings. ER prepared the first draft of the manuscript, which was iteratively completed and revised by both authors. Both authors read and approved the final manuscript.

## Pre-publication history

The pre-publication history for this paper can be accessed here:

http://www.biomedcentral.com/1471-2458/13/9/prepub

## Supplementary Material

Additional file 1 **In-depth responses, classified by health issue.** This table provides the validated written summaries of 15 in-depth interviews conducted with individuals that have applied the GRADE approach in the context of systematic reviews or guidelines in the field of public health. Click here for file
