# Supplementary material for: Current experience with applying the GRADE approach to public health interventions: an empirical study
Source: BMC Public Health. 2013 Jan 8;13:9. doi: 10.1186/1471-2458-13-9 (PMC3546302; doi:10.1186/1471-2458-13-9)
Supplement: Additional file 1 — In-depth responses, classified by health issue. This table provides the validated written summaries of 15 in-depth interviews conducted with individuals that have applied the GRADE approach in the context of systematic reviews or guidelines in the field of public health. [file 1471-2458-13-9-S1.docx]

**Additional files**

In-depth responses, classified by health issue

| **Health issue** | **Specific example(s)** | **Rating of GRADE experience** | **Specific issues (positive or negative)** | **Contact** |
| --- | --- | --- | --- | --- |
| HIV/AIDS | WHO recommendations on prevention and treatment of HIV and other sexually transmitted infections among men who have sex with men and transgender people | GRADE applied without significant challenges | - GRADE worked well but faces limitations when a range of population-focused approaches is assessed, i.e. when the guideline concerns a variety of approaches (rather than a single intervention) - Agreement on outcomes and a clear distinction between individual- and group-level outcomes is critical   - Distinction between final outcomes *versus* surrogate outcomes in grading is not clear-cut   - Individual interventions offered on demand with a focus on individual-level outcomes (e.g. earlier identification of HIV infection, reduced morbidity and mortality) *versus* proactive implementation of the same interventions as part of public health programmes with a focus on group-level outcomes (e.g. reduced community viral load, reduced HIV transmission and incidence) may yield different grading results - GRADE methodology includes useful flexibilities - GRADE for diagnostic purposes needs to be refined | HIV/AIDS Department, WHO |
| Tuberculosis | WHO guidelines for the programmatic management of drug-resistant tuberculosis | GRADE applied with minor challenges | GRADE has improved the quality of guideline development and no alternative is proposed. Based on experience with updating the guidelines on drug-resistant TB:   - GRADE worked reasonably well for observational studies, which are the source of most of the available evidence on treating patients with drug-resistant TB. It proved less apt at capturing the assumptions and methods behind modelling and cost-effectiveness studies. - Requirement for contextual evidence (eg. case series) and expert opinion to put things in perspective but no structured format for capturing these factors beyond expert decision tables. - Beyond the benefits/harms to the individual patient, an intervention may have positive externalities at a population-level. This public health perspective is not always well captured in the outcomes of studies (e.g. effect of successful TB treatment on secondary transmission) or in the considerations used in deriving a recommendation. One additional consideration in going from evidence to recommendations could be “Public health benefit”. - Some experts in the panel felt that, in some cases, the quality of evidence could have been upgraded based on analogy from treatment experience among other TB patients (e.g. by applying the experience of antiretroviral treatment use among TB/HIV patients to drug-resistant TB patients). | Stop TB Department, WHO |
| Nutrition | Various WHO guidelines on micronutrients | GRADE applied without significant challenges | - The GRADE approach is conservative in that the overall assessment of the quality of evidence is based on the lowest-rated body of evidence. The GRADE approach also encourages the contextualisation of the evidence during the process of developing recommendations by considering values and preferences, balance between benefits and harms, feasibility and cost associated with the intervention. - Panel members frequently struggle to translate the quality of evidence into the strength of a recommendation. - The GRADE methodology is not intuitive, particularly to those who are not familiar with a systematic assessment of the evidence. Guideline members require training to understand and be able to apply the GRADE methodology. | Department of Nutrition for Health and Development, WHO |
| Health systems | Pharmaceutical reference and index pricing policies (SUPPORT summary) | GRADE applied with minor challenges | - GRADE works but application to health systems interventions can be difficult - The inclusion of effect estimates can be challenging for health systems interventions and the Summary of Findings table needs to be adapted to accommodate narrative measures of impact. - Sometimes the quality of evidence changes based on other considerations, e.g. in a guideline on pricing policies interrupted time series studies were initially graded as low based on the GRADE criteria but then upgraded to moderate after reconsidering risk of bias. - Need for standardised Summary of Findings tables for narrative measures of impact and need to expand the Help option in GRADE and to provide a collection of frequent errors or good-practice examples. | SUPPORT network |
| Health systems | Conditional cash transfers and uptake of health interventions (SUPPORT summary)  Pay-for-performance and quality of health care (SUPPORT summary)  Expanding the role of outpatient pharmacists (SUPPORT summary)  Prompting Physicians in preventive care (SUPPORT summary) | GRADE applied with minor challenges | - GRADE works but application to health systems interventions can be difficult - Multiplicity of outcomes: Health systems interventions are characterised by multiple outcomes for each comparison. It is difficult to lump these in categories (e.g. utilisation of health services) that allow you to rate the quality of evidence in a more user-friendly way. - Effect measure: Rather than using a quantitative measure of effect, in many cases a "general" narrative measure of impact is the only option. Making judgements about GRADE criteria such as inconsistency or imprecision is cumbersome and difficult. - Often limited quantity of evidence makes judgements about publication bias very difficult. - Difficulty in applying criteria such as inconsistency, imprecision and publication bias in a reliable way. - Need to produce guidance on how to use GRADE and apply GRADE criteria with narrative evidence synthesis. Including examples of common errors and good practice. | SUPPORT network |
| Child health | Various WHO guidelines on community-based newborn care | GRADE applied with minor challenges | - GRADE was applied to individually and cluster-randomised as well as quasi-randomised studies; the evidence base for these guidelines did not include observational studies - GRADE was perceived to be too subjective and, as a result, the approach was refined to make the criteria more objective and specific by   - re-definition of selected criteria (e.g. consistency)   - awarding points for all sub-criteria   - deciding on whether criteria apply when studies account for >50% or >75% of total weight of evidence and,   - defining exactly how final score of study is to be derived - Example of weighting/points scheme used for criterion “limitations in methods”   - five sub-criteria, i.e. design, allocation concealment, blinding or other approaches to reduce measurement bias, ITT analysis or cluster adjustment or adjustment for confounding (where applicable), loss-to-follow up   - assignment of 0, -0.5 or -1 points that can be lost for each sub-criterion - Non-randomised trials were initially grouped with randomised trials and then downgraded | Department for Child and Adolescent Health, WHO |
| Child health | WHO guidelines on HIV and infant feeding  WHO guidelines on the management of children with severe malnutrition | GRADE applied with major challenges | - GRADE provides a good process for clearly and systematically assessing and documenting the quality of evidence and for bringing more objectivity into the guideline development process - These examples did not follow the CAH-adaptation of GRADE described above. In comparison, there were few randomised trials contributing high quality evidence and some data originated from basic physiology and basic science research (e.g. physiological energy cost for synthesising protein): GRADE is perceived to be a mechanical approach but represents principles for handling evidence that can and should be applied in a ways that reflect the recommendations in question and the nature of evidence that is available – “life does not fit into square boxes” - GRADE does not protect against subjectivity: when used too rigidly, there is a danger of omitting important information   - Potentially reduction of evidence to effectiveness outcomes with little interpretation of methods and analyses   - Loss of important information about context/qualitative/explanatory issues   - Assessment of benefits vs harms is very subjective which, in a positive way, can be used to consider important qualitative issues but, in a negative way, may also circumvent such issues   - When RCTs are not available it becomes difficult to include and benefit from non-research experiences and reports   Examples where GRADE quality of evidence did not appropriately reflect confidence in evidence:  Application of generally accepted nutritional principles to HIV-positive children   - Large body of evidence derived for HIV-negative children; there is no reason why these should not equally apply to HIV-positive children (analogy) - The guideline panel felt that the evidence was sufficiently indirect to require downgrading   Application of fundamental physiological principles   - Number of calories required to manufacture new muscle derived from laboratory experiments/basic science - downgraded as this type of evidence does not fit RCT classification   GRADE could be used better within WHO   - Asset: do not use it as a “policing aid” by GRC but as an asset that can help with the guideline development process - Training/guidance: chair of guideline group has a critical role and does have to be very skilled in navigating the process; more guidance on how to manage GRADE process is needed - Nomenclature: “low” implies poor quality/badly conducted studies which can be both misleading and damaging when used with policy-makers | Department of Maternal, Newborn, Child and Adolescent Health, WHO  Department of Nutrition for Health and Development, WHO |
| Vaccination | Various WHO guidelines on vaccination | GRADE applied with minor challenges | - GRADE works with more specific instructions/clarifications on its application and minor adjustments to the process of scoring the quality of evidence - Stakeholders do not use GRADE tables - Put GRADE tables on the web but do not include them with vaccine position papers - Cite GRADE score but focus on confidence in the estimate of effect rather than the “low” versus “high” terminology - To keep the guideline process feasible, GRADE is only applied to the most important questions in the evidence review process - GRADE discriminates against observational studies, independent of specific epidemiological study design (e.g. self-controlled case series) - Systematic reviews often do not consider all of the data that are relevant to the assessment of vaccine efficacy and safety - Relevant and important data are excluded or downgraded despite their importance in the decision-making process - Low rankings (where they do not appropriately reflect the quality of the evidence base, e.g. lack of evidence on side-effects of vaccination) may fuel the anti-vaccination agenda - Clarifications and modifications: - Study design (i.e. self-controlled case series enter as moderate evidence) - Studies should not be repeatedly penalised for limitations already factored into their starting score - New upgrading criterion ability of design to control for confounding and avoid biases and consistency across settings and studies (i.e. different settings, extended periods of time, different investigators) This comes under mitigated bias and confounding - Specific cases discussed at GRADE Working Group meeting in Geneva: - Vaccine and intussusception: low-quality evidence is upgraded using modified GRADE criteria (e.g. +2 for polio for ability of design to control for confounding and consistency across settings and investigators (Vaccine article) (not agreed with GRADE Working Group) - Upgrading for population-based impact (i.e. increased levels of risk reduction with increased vaccine coverage and reversal of effect associated with decreased level of vaccine coverage) under dose-response criterion (agreed with GRADE Working Group) | Strategic Advisory Group on Immunisation, WHO |
| Mental health | Guidelines for mental, neurological and substance use disorders within WHO Mental Health Gap Action Programme | GRADE applied with major challenges | - GRADE can be applied to guidelines on clinical interventions but was not used for prevention and diagnostic guidelines, as observational evidence is not available in GRADE-compatible format, i.e. systematic reviews of observational studies are typically not available and the meta-analytical process of pooling results from observational studies may not be methodologically appropriate - GRADE process can/should be further improved in relation to   - (i) inclusion of non-randomised evidence and evidence that cannot be meta-summarised,   - (ii) reproducibility and internal consistency,   - (iii) choice of outcome measures, and   - (iv) guidance on how to better consider value judgements, resource use and feasibility - Risk of omitting evidence from non-randomised studies, when both randomised and observational evidence is available, which may create a serious bias towards high-level quality of evidence in the guideline process - No explicit guidance for selection of outcome measures when multiple measures are available (e.g. rating scales for mental health), which leads to inconsistencies and bias - GRADE process shows problems with reproducibility and consistency, as grading criteria are sometimes concerned with the quality of a group of studies (e.g. limitations, indirectness) and sometimes with the meta-analytic process of data aggregation (e.g. inconsistency, publication bias); both groups of criteria have equal weight, which is not necessarily the best way forward for public health recommendations - Need for more pragmatic instructions on how to grade quality of evidence, especially when a systematic review includes more than 100 studies - Ad hoc process (rather than clearly described methodology) for taking into consideration values, preferences and feasibility issues in developing recommendations - GRADE offers little guidance on applicability when much evidence is *from* developed countries *for* developing countries judged through (i) directness, (ii) characteristics of delivery agents/systems and (iii) availability of implementation studies - Need to explore how publication bias can be judged in a more reliable way, as a funnel plot is rarely available and as its interpretation is not always straightforward | Evidence, Research and Action on Mental and Brain Disorders, WHO |
| Environmental health | WHO indoor air quality guidelines: household fuel combustion (ongoing) | GRADE applied with major challenges | - Three main reasons for why applicability of GRADE to environmental health interventions shows limitations - RCTs are difficult to conduct for many environmental health interventions with quasi-experimental designs often representing the only feasible alternative - Criteria within GRADE to grade quality of observational studies are too limited - GRADE does not allow for an adequate appreciation of alternative sources of evidence (e.g. analogies from similar health risks) - Diversity of epidemiological and non-epidemiological evidence along environmental health pathway - Source-emissions link (i.e. stove testing, engineering) - Emissions-concentrations/exposure link (i.e. air pollution measurements, modelling based on physical principles) - Concentrations/exposure-health link (i.e. epidemiology, exposure-response) - Multiple short-term and long-term health outcomes | Department of Public Health and Environment, WHO |
| Health systems | WHO guidelines on the retention of rural health workers | GRADE applied with minor challenges | - Health systems research is characterised by a varied evidence base that is dominated by descriptive studies (e.g. case studies, observational studies) rather than intervention studies (e.g. RCTs) - Need to use surrogate outcomes rather than morbidity/mortality for health systems interventions - Very large influence of context on effectiveness of health systems interventions needs to be carefully examined - Health systems studies often do not report estimates of effect (e.g. OR, RR), making the application of the GRADE criteria challenging - Contradiction between GRADE assessment and expert judgement may be in part determined by limited high-quality evidence available, e.g. moderate-quality evidence based on 2-3 studies that financial incentives are not effective in the long term but country-specific experience that financial incentives can be effective in the short-term - Need to develop clearer guidance on how to use criteria other than evidence of effectiveness (i.e. values and preferences, feasibility and resource use) in deriving recommendations - Currently also working on guidelines on education and training of health workers, where a mixed approach of guidelines and policy briefs (rather than just guidelines) may be applied | Department of Health Policies, Systems and Services, Human Resources for Health unit, WHO |
| Environmental health | Housing improvements for health and associated socio-economic outcomes (Cochrane systematic review, ongoing) | GRADE currently being tested | - Difficulty in incorporating evidence from non-randomised studies into the GRADE process when there are some RCTs, but the weight of evidence comes from non-randomised studies. - Difficulty in assessing study limitations across studies. It is hard to predict how particular risk of bias items may have affected the outcomes making it hard to choose items of most importance. The sensitivity to differentiate between different types of non-randomised studies is very limited. - Need to include range of outcomes and measures under one “umbrella outcome” requires a decision on whether to downgrade for indirectness or not. - Studies related to a given public health intervention are usually characterised by much heterogeneity in interventions, comparisons and outcomes, which requires a decision on whether to downgrade for inconsistency or not. Regarding explained versus unexplained inconsistency, the studies were divided into (i) categories of intervention, (ii) context and (iii) timescale, yet some heterogeneity remained which is almost always the case due to the broad scope of these types of review questions. - Applying GRADE to narrative summaries is challenging. In particular, lack of a pooled effect size across included studies limits assessment of the following GRADE criteria:   - Inconsistency (we examined differences in effect type, direction of effect and effect estimates between studies as much as possible)   - imprecision (random error)   - magnitude of effect   - publication bias (often there are not enough studies for an informative funnel plot) | Public Health Group, Cochrane Collaboration |
| Screening | Guidelines on cervical cancer screening [1]  Guidelines for hepatitis B screening among immigrants and refugees [1]  Guidelines for latent TB screening among immigrants and refugees [2]  Guidelines on breast cancer screening [3] | GRADE applied with minor challenges | - Deciding on the best evidence for deriving an estimate of benefits and harms for patient important outcomes can be difficult for complex interventions when no bridging RCTs or controlled observational studies are available (e.g. need to consider ecological studies vs. cohort studies) - Time and methodological challenges in finding, selecting and synthesising evidence in systematic reviews of observational studies. As many different sources of low-quality observational evidence do not necessarily increase the overall quality of evidence (confidence in effects), modelling may be a promising route to help improve effect estimates but modelling evidence comes with its own significant risk of bias challenges. - Need to develop a systematic approach within GRADE to assess the quality of modelling studies with options for upgrading - Complex interventions (e.g. detection-follow up-treatment continuum in screening) tend to be reduced to simple interventions such as treatment element (ignoring the detection, follow-up elements) in systematic reviews and this requires a decision whether the evidence should be downgraded for indirectness or not (e.g. high quality of evidence for latent TB screening among Canadian immigrants is based on a systematic review of treatment efficacy and not downgraded for indirectness) - Applying GRADE to clinical prevention will often result in low quality evidence ratings and this can result (although not necessarily) in weak recommendations. Weak recommendations could mean that these services are not funded in certain jurisdictions with potentially significant implications for interventions such as vaccination where there is a need for herd immunity (society contribution) to improve effectiveness. | Canadian Task Force on Preventive Health Care, Canadian Guidelines for Immigrant Health |
| Reproductive health | Guidelines on prevention and control of cervical cancer (ongoing) | GRADE applied with major challenges (health education), minor challenges (remaining parts of guideline) | - Advantages of GRADE approach:   - very systematic and rigorous about question formulation, search for and appraisal of evidence - Disadvantages of GRADE approach:   - Very resource-intensive, time-consuming process to obtain a high level of precision   - Added value beyond other evidence-based approaches is not certain   - Not suitable for complex interventions - GRADE is well suited to clinical interventions, need to use a more flexible alternative for complex interventions that is more inclusive of broader literature - Guideline addresses a large spectrum of questions including (i) primary prevention (e.g. condom use, health education, HPV vaccination), (ii) early prevention (e.g. screening through PAP test, HPV testing, cytology, visual inspection and subsequent treatment of lesions), (iii) treatment of invasive cancer and (iv) palliative care among the general population as well as HIV+ women - Each of these intervention complexes includes several different intervention options alone and in combination, e.g. “screen and treat” covers multiple combinations of different screening and treatment - Obtaining a direct estimate of intervention impact on morbidity/mortality is challenging with long-term, delayed outcomes such as cancer - Complex interventions are critically dependent on context, e.g. low-cost technology of visual inspection for screening is not the most effective strategy available but the only feasible option in low-resource settings - GRADE is not adapted to health education interventions (e.g. health education to delay first sexual intercourse), where high-quality studies about the intervention are either (i) not available with a recommendation relying on a very low-quality consensus statement, (ii) highly dependent on context (e.g. cultural issues around sexual behaviour) and thus not transferable - PICO may force guideline development group to be artificially restrictive in the literature search, where the available literature often does not correspond to the PICO question and related evidence does not meet the inclusion criteria - Very costly process: 1^st^ edition of guideline (2005) cost approx. US$ 500 000.-; one chapter of 2^nd^ edition of guideline (ongoing) approx. US$ 600 000.- | Department of Reproductive Health and Research, WHO |
| Child health | Canadian physical activity and sedentary guidelines for the early years | GRADE applied with major challenges | - Challenges encountered in relation to public health guidelines - Selected GRADE terminology (e.g. patients, clinicians) does not apply to public health - The use of “weak recommendations” is perceived to be problematic - Difficulties in applying the quality of evidence framework to:   - Body of evidence consisting of observational studies   - Devaluation of cross-sectional studies led to reliance on small often non-representative evidence base   - Distinction between efficacy and effectiveness - Problems with GRADE summary of findings tables for narrative summaries:   - insufficient as an accurate summary of the underlying literature   - difficult and cumbersome to produce where meta-analysis is not possible | The Physical Activity Measurement and Guidelines Steering Committee, Canadian Task Force on Preventive Health Care |

It should be noted that, while GRADE experiences are associated with specific departments/organisations, respondents alone are responsible for the views expressed; these views do not necessarily represent the decisions or policies of the organisations they are affiliated with.

**References:**

[1] Pottie K, Greenaway C, Feightner J, Welch V, Swinkels H, Rashid M, et al. Evidence-based clinical guidelines for immigrants and refugees. CMAJ 2011; 183(12): E824-E925.

[2] Greenaway C, Sandoe A, Vissandjee B, Kitai I, Grunder D, Wobewer W, et al. Tuberculosis: evidence review for newly arriving immigrants and refugees. CMAG 2011;183(12):E939-E951.

[3] The Canadian Task Force on Preventive Health Care. Recommendations on screening for breast cancer in average-risk women aged 40–74 years. CMAJ 2011;183(17):1991-2001.
